# Timing and Operating Mode Design for Time-Gated Fluorescence Lifetime Imaging Microscopy

**DOI:** 10.1155/2013/801901

**Published:** 2013-06-24

**Authors:** Chao Liu, Xinwei Wang, Yan Zhou, Yuliang Liu

**Affiliations:** Optoelectronic System Laboratory, Institute of Semiconductors, CAS, Beijing 100083, China

## Abstract

Steady-state fluorence imaging and time-resolved fluorescence imaging are two important areas in fluorescence imaging research. Fluorescence lifetime imaging is an absolute measurement method which is independent of excitation laser intensity, fluorophore concentration, and photobleaching compared to fluorescence intensity imaging techniques. Time-gated fluorescence lifetime imaging microscopy (FLIM) can provide high resolution and high imaging frame during mature FLIM methods. An abstract time-gated FLIM model was given, and important temporal parameters are shown as well. Aiming at different applications of steady and transient fluorescence processes, two different operation modes, timing and lifetime computing algorithm are designed. High resolution and high frame can be achieved by one-excitation one-sampling mode and least square algorithm for steady imaging applications. Correspondingly, one-excitation two-sampling mode and rapid lifetime determination algorithm contribute to transient fluorescence situations.

## 1. Introduction

Fluorescence imaging techniques are widely used in biomedical applications. Fluorescence can be classified in autofluorescence and extrinsic fluorescence. Autofluorescent substances in biological tissues like several amino acids, NADPH, and flavin can be utilized for fluorescence imaging [1]. External fluorescence is generated by the combination of fluorophore and relative target molecule. Common fluorophores include organic dyes, fluorescent protein, quantum dots, and long lifetime metal-ligand complexes (MLCs). Organic dyes such as Cy5, rhodamine series dyes have been fully used for fluorescence imaging experiments. Their lifetimes range from ns level to hundreds of ns. Fluorescent proteins are a powerful tool for biomedical imaging. One of the most famous delegates is GFP. Quantum dots can be excited by laser from 300 nm to 1000 nm according to different types and they express lifetimes between ns to hundred ns [2, 3]. MLCs possess long stokes shift and long lifetime from *μs* to ms level. One important application is intracellular oxygen molecule imaging [4, 5].

Fluorescence will decay after being excited according to a specific pattern which is often fitted by negative exponent functions [6]. Fluorescence decay process can be assorted to steady-state and time-resolved (transient) categories [7]. Target molecule barely changes during steady fluorescence decays. Differentiation of Cancer lesion area from normal tissues is one representative application of steady fluorescence measurement. Time-resolved measurement shows the dynamic process of the target molecule during imaging. Important biochemical processes like protein folding, carbon fixation in photosynthesis, and some FRET processes all take place in ms to several hundred ms [8].

Fluorescence intensity [9], polarization [10], and lifetime which carry the microenvironment changing information of the target molecule can all be utilized for imaging. Fluorescence lifetime *τ* is definite as the time when fluorescence intensity attenuates to the 1/*e* of the initial intensity. Fluorescence lifetime is an absolute value which is independent of excitation laser intensity, fluorophore concentration, and photobleaching. Furthermore, fluorescence lifetime can be applied to distinguish the molecules which are indiscernible using fluorescence intensity imaging methods. Flavin adenine dinucleotide (FAD) and flavin mononucleotide (FMN) share the same optimum excitation wavelength and emission wavelength; however, the different lifetimes of the two provide one solution for this dilemma [11].

Compared with fluorescence intensity microscopy (FIM), fluorescence lifetime imaging microscopy (FLIM) has the advantage that it is less influenced by dye or target molecule concentration, photo bleaching, and excitation laser intensity. Typical methods to realize FLIM include time-gated FLIM, time correlated single photon counting (TCSPC), and Frequency Modulation.

As the needs for high frame frequency and high-resolution microscopy increase, the low-cost advantage of frequency modulation FLIM becomes less dominant compared with that of temporal FLIM including time-gated FLIM and TCSPC.  Table 1 compares the time resolution and imaging frame of the two mainstream temporal domain methods. Mature TCSPC products can be provided by many institutes and companies. TCPSC-FLIM can achieve very high temporal resolution, as in Table 1. However, due to its scanning processing pattern, TCPSC-FLIM cannot provide as high frame frequency as the one that time-gated FLIM can realize. TCPSC-FLIM is suitable for steady fluorescence imaging which requires high time resolution such as cancer detection. As for important intraocular transient process imaging such as Ca^2+^ movement and protein fusion, high frame frequency time-gated FLIM can supply a potential method for these applications. Imperial College London, UC Davis [12], University Michigan [13], and Utrecht University [14] have applied time-gated FLIM to endoscope FLIM clinical surgery, in vitro or in vivo imaging, and so forth. Important areas of time-gated FLIM focus on improvement of temporal and spatial resolution and improvement of image speed to satisfy the need for transient fluorescence imaging. Time-gated FLIM can be a complimentary method for FIM to provide a clearer and faster way of tracking the microcosms.

In all, time-gated FLIM can be performed for steady and transient fluorescence imaging applications.

## 2. Methods

**Figure 1:**
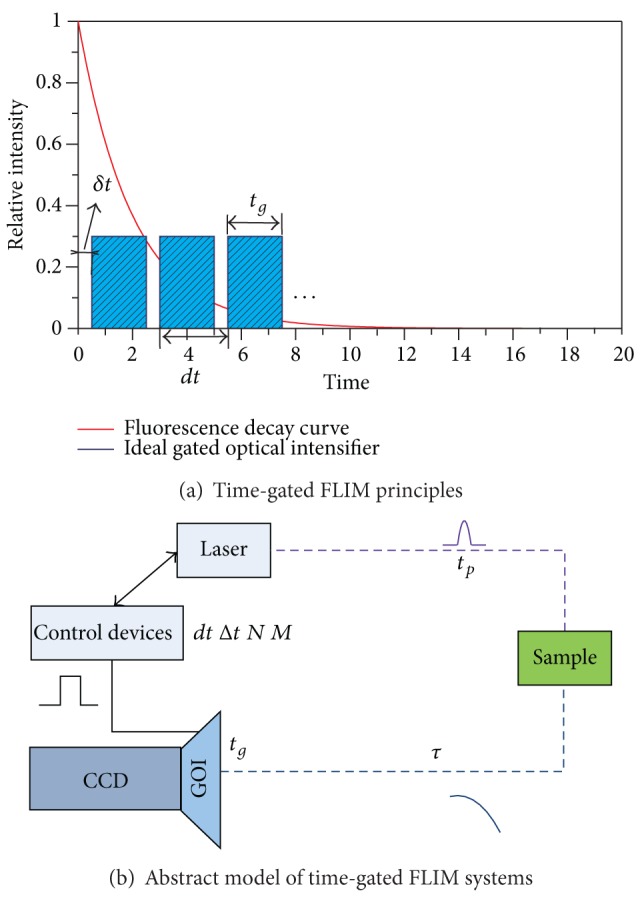
Abstract system model and temporal parameters. (*t*_*p*_ shows laser pulse, *τ* stands for sample lifetime, and *t*_*g*_ means gate width of gated optical intensifier (GOI), *N*, *dt*, Δ*t*, *M* and *δt* represent number of gates opened in one excitation, gate interval of *N* gates, delay step of different excitations, number of excitations, and the interval between laser excitation and opening of the first gate, resp.).

Time-gated FLIM acquires different images at different delays according to laser excitation (Figure 1(a)), and thus lifetime images are computed through specific inversion algorithm [17]. Due to different types of time-gated FLIM systems, a simple abstract model was given to explain how the system works. Sample is excited by short pulse laser. Fluorescence images are coupled into ICCD by light path design. Control devices realize synchronization of laser and ICCD, and thus gates open at different delay according to laser. Lifetime image will be computed using processed intensity images under certain algorithms.

Lifetime algorithm was designed on Matlab R2007b, Math Works. Timing was realized using a homemade Time Control Unit (TCU) based on ARM (STM32F103VE, ST), FPGA (XC3S400, Spartan-3, Xilinx), and ISE platform [12]. TCU generates two timing sequences to be utilized to trigger pulse laser and ICCD separately. The main technical parameter of the TCU can be seen in Table 2.

## 3. Results and Discussion

### 3.1. System Control and Temporal Parameters

To acquire different intensity images at different delay in order to compute lifetime image, it is crucial to control the ICCD and laser synchronously. FLIM system can be built up by inner trigger control [18], external trigger control [19], or optical delay control [20] according to different types of ICCD and laser utilized.  Figure 1(b) shows an abstract system model of different control methods and important temporal parameters involved. *t*_*p*_ shows laser pulse, *τ* stands for sample lifetime, and *t*_*g*_ means gate width of gated optical intensifier (GOI). Moreover, *N*, *dt*, Δ*t*, *M*, and *δt* which represent number of gates opened in one excitation, gate interval of *N* gates, delay step of different excitations, number of excitations and the interval between laser excitation and opening of the first gate, respectively determine different operating modes. *δt* should be short enough to collect enough fluorescence to increase the first delay signal which is crucial for lifetime computing but not too short to saturate ICCD because of the strong laser [21]. In addition, *δt*, *dt*, and Δ*t* are defined according to the rising edge of located gate. Gated function can be summarized as a universal equation after the definition of these important temporal parameters. Timing design was based on this equation for steady and transient operating-mode of time-gated FLIM. *N*, *dt*, Δ*t*, *M*, and *δt* can either be fixed or variable according to different operating modes:

(1) $$\begin{array}{c} g\left(t\right)=\overset{M-1}{\underset{j=0}{\sum }}\;\overset{N-1}{\underset{i=0}{\sum }}w\left(t+idt_{\left(i\right)}+j\Delta t_{\left(j\right)}+\delta t_{\left(j\right)}\right),\\ w\left(t\right)=\{\begin{array}{cc} 1, & 0\leq t\leq t_{g},\\ 0, & \text{otherwise}. \end{array} \end{array}$$

### 3.2. Lifetime Computing Algorithm Design

**Figure 2:**
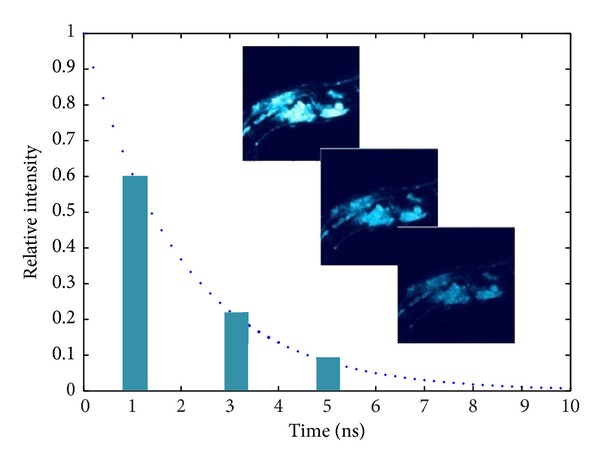
Nematode GFP-gated intensity images.

Lifetime computing algorithm development can be seen in our former proceeding papers [6]. Parts of gated intensity images of a nematode containing GFP provided by Becker & Hickl GmbH using TCSPC method [15] can be seen in Figure 2. Original images contain a series of 8 images recorded at 1 ns to 8 ns. To realize high-speed computing under transient fluorescence circumstances, lifetime will be computed by two intensity images before next excitation under rapid lifetime determination (RLD) method. Lifetime is computed based on (2), where the interval between two images equals gate interval *dt*, *I*_1_ and *I*_2_ represent image intensity:

(2) $$\begin{array}{c} \tau =\frac{dt}{\ln\left(I_{2}/I_{1}\right)}. \end{array}$$

**Figure 3:**
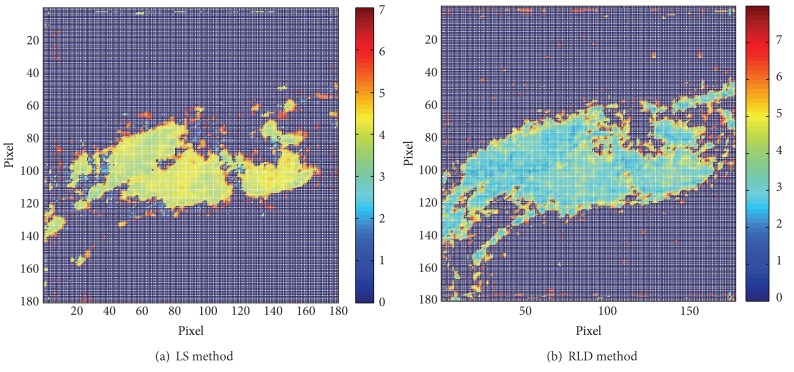
Stimulated result of nematode gated image.

For steady fluorescence lifetime computing, a least square algorithm is sufficient for a precise inversion. Fluorescence decay pattern is inverted per pixel through least square fit, and thus lifetime is computed by its definition in Section 1. Both RLD method and LS method results based on former GFP images can be seen in Table 3 and Figure 3.

### 3.3. System Operating Mode Design

Timing design was based on (1). In this paper, two timings are designed for steady-state and transient fluorescence recording.

**Figure 4:**
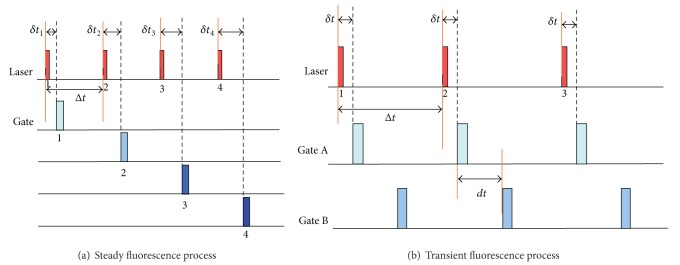
Operating mode and timing design for time-gated FLIM.

For steady-state fluorescence, an operating mode and timing are designed in Figure 4(a). For each excitation, only one intensity image will be recorded (*N* = 1) to accommodate with low frame frequency ICCD, namely, one-excitation one-sampling mode. *M* and Δ*t* are determined by laser RF. Δ*t* is fixed while *δt* is variable. This timing can be explained by (3) which is degenerated from (1):

(3) $$\begin{array}{c} g{\left(t\right)}_{s}=\overset{M-1}{\underset{j=0}{\sum }}w\left(t+\Delta t+\delta t_{\left(j\right)}\right). \end{array}$$

Delay among each excitation and opening of its gate *δt* during one sampling determines delays of each intensity image. Imaging inversion speed is not as important as transient fluorescence lifetime in steady case; therefore, algorithms with high accuracy will be more suitable for steady fluorescence lifetime applications. In which case, LS method can be utilized to process different delay images after the former procedure.

However, for transient fluorescence process such as Ca^2+^ movement which usually occurs in a time order of hundreds or even tens of milliseconds, high-frequency imaging frame and high-speed lifetime computing algorithm are highly emphasized. In which case, Figure 4(b) provides a feasible mode and timing to realize transient fluorescence recording. Several gates (*N* > 1) will be opened in one excitation at the same time, and thus *N* intensity images are at different delays recorded simultaneously, namely, one-excitation multiple-sampling mode:

(4) $$\begin{array}{c} g{\left(t\right)}_{t}=\overset{M-1}{\underset{j=0}{\sum }}\;\overset{N-1}{\underset{i=0}{\sum }}w\left(t+idt+j\Delta t+\delta t\right). \end{array}$$

Main parameters in (4) are all fixed. Here, an embodiment is shown in Figure 4(b) where *N* = 2 and Gate A and Gate B are opened in one excitation with a specific delay (*dt*). RLD algorithm utilizing two intensity images was developed to realize high-speed lifetime image inversion. Lifetime image can be inverted before next excitation. Two images acquired simultaneously based on fibre bundles gave a method to realize this timing [20]. Therefore, lifetime can be computed in one single excitation since two synchronous images are acquired and processed in real time. Every CCD frame will be fully exploited for lifetime image acquired with the coordination of laser RF and CCD frame by this timing and operating mode.

## 4. Conclusion

To sum up, aiming at steady and transient fluorescence lifetime imaging, we present feasible operating modes and lifetime computing algorithms (Table 4). High resolution and high frame can be achieved by one-excitation one-sampling mode and least square algorithm for steady imaging applications. Correspondingly, one-excitation two-sampling mode and rapid lifetime determination algorithm contribute to transient fluorescence situations. The universal timing equation (1) can be used for another application after certain temporal parameters changed. The abstract model (Figure 1(b)) can provide a basis for time-gated FLIM system buildup.

## Figures and Tables

**Table 1:** Parameters comparison of different FLIM methods.

	Time-gated FLIM	TCSPC [15]	Frequency modulation
Time resolution	50 ps [13]	1 ps	—
Frame frequency	100 Hz [14]	10 Hz	1 Hz [16]

**Table 2:** Technical parameters of homemade TCU.

Repetitive frequency	Minimum delay step	Minimum pulse width	Delay revise precision
1 Hz–100 kHz	500 ps	2 ns	150 ps

**Table 3:** Comparison of exponential pattern model and polynomial model.

	Average lifetime (ns)	Standard deviation	Computing time (s)*
RLD	5.27	0.79	0.04
LS	4.30	1.32	36.8

*Intel Pentium T3200 @ 2 GHz, RAM 2 GB, Matlab R2007b, Math Works.

**Table 4:** Operating mode and lifetime computing algorithm for different applications.

	Operating mode	Lifetime computing algorithm
Steady fluorescence	One-excitation one-sampling mode	LS
Transient fluorescence	One-excitation two-sampling mode	RLD
